# Improving Resolution of Dual-Comb Gas Detection Using Periodic Spectrum Alignment Method

**DOI:** 10.3390/s21030903

**Published:** 2021-01-29

**Authors:** Haoyang Yu, Qian Zhou, Xinghui Li, Xiaohao Wang, Xilin Wang, Kai Ni

**Affiliations:** 1 Division of Advanced Manufacturing, Tsinghua Shenzhen International Graduate School, Tsinghua University, Shenzhen 518055, China; yu-hy16@mails.tsinghua.edu.cn (H.Y.); zhou.qian@sz.tsinghua.edu.cn (Q.Z.); li.xinghui@sz.tsinghua.edu.cn (X.L.); wang.xiaohao@sz.tsinghua.edu.cn (X.W.); 2 Engineering Laboratory of Power Equipment Reliability in Complicated Coastal Environments, Tsinghua Shenzhen International Graduate School, Tsinghua University, Shenzhen 518055, China; wang.xilin@sz.tsinghua.edu.cn

**Keywords:** dual-comb spectroscopy, molecular absorption spectroscopy, gas detection, error correction, noise suppression

## Abstract

Dual-comb spectroscopy has been an infusive spectroscopic tool for gas detection due to its high resolution, high sensitivity, and fast acquisition speed over a broad spectral range without any mechanical scanning components. However, the complexity and cost of high-performance dual-comb spectroscopy are still high for field-deployed applications. To solve this problem, we propose a simple frequency domain post-processing method by extracting the accurate position of a specific absorption line frame by frame. After aligning real-time spectra and averaging for one second, the absorbance spectrum of H^13^C^14^N gas in the near-infrared is obtained over 1.1 THz spectral range. By using this method, the standard deviation of residual error is only ~0.002, showing great agreement with the conventional correction method. In addition, the spectral resolution is improved from 13.4 GHz to 4.3 GHz compared to direct spectrum averaging. Our method does not require a specially designed common-mode suppression comb, rigorous frequency control system, or complicated computational algorithm, providing a cost-effective scheme for field-deployed Doppler-limited spectroscopy applications.

## 1. Introduction

The optical frequency comb (OFC) consists of an array of discrete modes in the optical frequency domain. The absolute frequency of each mode $\upsilon_{n}$ is determined by two radio frequency (RF) freedoms, i.e., repetition frequency $f_{r}$ and offset frequency $f_{0}$, as $\upsilon_{n}=f_{0}+nf_{r}$ [1,2,3]. The spectral coverage of OFC can be expanded from extreme ultraviolet to infrared and terahertz domain via self-phase modulation and nonlinear conversion [4,5,6,7]. As a coherent broadband laser source, OFC is extremely attractive for numerous spectroscopic applications [8].

To separate the optical modes and recover the encoded spectral information, virtually imaged-phased-array (VIPA) [9], Fourier transform spectroscopy (FTS) [10], and dual-comb spectroscopy (DCS) [11] are mostly employed for direct comb-based spectroscopy. DCS introduces a second local oscillator (LO) comb with slightly different repetition frequency difference to replace the dispersion device in VIPA or mechanical scanning components in FTS. Due to the unique capability of obtaining high resolution, high sensitivity, broad spectral range, and fast measurement speed simultaneously, DCS has drawn considerable attention since its first implementation [12] and brought significant improvement for applications such as molecular absorption spectroscopy [13], coherent anti-Stokes Raman spectroscopy [14], multidimensional spectroscopy [15], time-resolved spectroscopy [16], ellipsometry [17], strain sensors [18], hyperspectral imaging [19], and ranging [20,21].

With the rapid development of stable, robust, and low-cost fiber mode-locked laser [22,23], DCS is gradually moving from the precise metrology laboratory to the field environment. Volatile organic compounds monitor [24], trace-gas source attribution [25], and vehicle carbon dioxide emissions [26] have been successfully demonstrated in the past few years, showing promise for replacing traditional sensing scheme. Despite high short-term stability for real-time spectral measurement [27], repetition frequency and offset frequency of fiber OFC is fairly sensitive to the environmental perturbation such as temperature drift, vibration, and pump noise [28], which would cause mutual coherence degradation and resolution loss. In addition, long term coherent averaging for obtaining higher sensitivity is also invalid, limiting the actual performance of a field-deployed DCS configuration.

To solve this problem, a tight lock of both repetition frequency and offset frequency has been first adopted [13,29]. However, this scheme requires bulk ultra-stable continuous wave (CW) laser reference and ultra-bandwidth actuator. Considering the significantly greater importance of relative stability between dual combs instead of absolute stability, more simplified DCS schemes have been proposed. These schemes can be divided into two branches. The first branch pursued to improve the natural coupling between two passive mode-locked lasers, several dual-comb shared single cavity light sources have been designed to suppress the common-mode noise [30,31,32]. This method is effective and low-cost, yet the tuning flexibility, stability, and robustness are still deficient compared to separate dual-comb configuration, which limits the hand-off running time and potential industrial perspective. The second branch recovered the mutual coherence via synchronous locking [33], adaptive sampling [34,35], real-time digital error correction [36], or self-referenced post-processing algorithm [37,38,39,40]. Among these methods, the self-referenced post-processing algorithm eliminates the CW reference and additional heterodyne interferometer, achieving the highest cost-effective for Doppler-limited spectroscopy. However, most self-corrected algorithms aim at correcting interferograms (IGMs) in the time domain, requiring relatively complicated calculation steps and rigorous limitations [39]. In fact, for actual spectroscopic applications such as gas detection, these time-domain correction processes are not necessary. Because the real-time spectrum of IGMs is of high fidelity with merely overall frequency drift [41,42], the spectrum alignment process has great potential to correct a dominated error in the frequency domain. In 2017, Kara et al. [43] proposed a spectrum alignment method based on global cross-correlation, proving the effectiveness of frequency domain correction. This method has high robustness but requires considerable computing resources, especially when pursuing high resolution and broad spectral range simultaneously. In addition, the absolute frequency calibration is based on bulk f-2f nonlinear interferometers in her demonstration.

In this paper, we propose a spectrum alignment method completely in the frequency domain for dual-comb gas detection. Our method only focuses on the center of gravity of the regional spectrum with low algorithm complexity and resource occupancy, making it easier to be implemented on a real-time hardware platform. Two passive mode-locked fiber lasers without complicated carrier-envelope offset frequency lock are used to interrogate H^13^C^14^N gas. Real-time transmittance spectra with 2 ms refresh time are obtained by fast Fourier transform, background removal, and alignment process. After 1 s averaging, the calculated spectrum is compared to the results by conventional digital error correction method to verify the effectiveness. Meanwhile, the resolution improvement effect is verified compared to direct spectrum averaging. Our method is simple and low-cost, offering an effective technique for field-deployed dual-comb applications.

## 2. Methods

**Figure 1:**
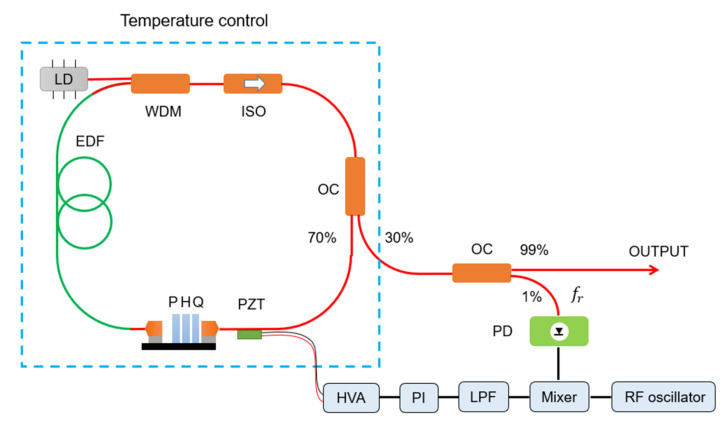
Fiber optical frequency comb (OFC) design. Red lines represent single-mode fiber paths, while black lines are electrical wires. LD: laser diode; EDF: erbium-doped fiber; WDM: wavelength division multiplexer; ISO: isolator; OC: optical coupler; Q: quarter-wave plate; H: half-wavelength plate; P: polarizer; PD: photodetector; LPF: low-pass filter; PI: proportional–integral controller; and HVA: high voltage amplifier.

The design of our home-made nonlinear polarization rotation (NPR)-based fiber OFC source is shown in Figure 1. Erbium-doped fiber (RightWave EDF80, OFS, Norcross, GA, USA) is pumped by a 1480 nm laser diode (FOL1437R50, FITEL, Japan) via a 1480/1550 nm wavelength division multiplexer. The polarization control module (OZ optics), containing quarter-wave plate, half-wavelength plate, and polarizer, serves as an artificial saturable absorber to achieve self-mode-locking. The fiber isolation ensures the operation direction of the ring cavity and the 70:30 coupler provided continuous laser output. The net dispersion of the oscillator cavity is ~−0.01 ps^2^ in the stretched-pulse regime. All the fiber components are basic non-polarization-maintaining optical communication devices (AFRs). The whole fiber mode-locked laser with ~8 mW average power is integrated into a temperature-controlled portable box, one percent of output power is collected by a photodetector (PDA05CF2, Thorlabs, Newton, NJ, USA) to control the repetition frequency by adjusting the length of low-speed piezoelectric actuators (AE0505D16F, Thorlabs, Newton, NJ, USA). The introduction of loose repetition frequency control (~10 Hz bandwidth) would not change the free-running nature of the mode-locked laser but guarantee long-term stability and better tuning flexibility. The center of the laser spectrum locates at ~1560 nm with ~20 nm full width at half maximum (FWHM), which could be further amplified and broadened to one octave by erbium-doped fiber amplifier and high nonlinear fiber [4].

**Figure 2:**
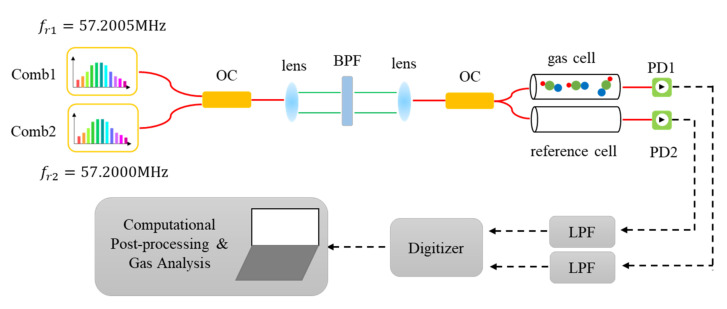
Experimental setup of dual-comb gas detection. Red solid lines represent single-mode fiber paths, green solid lines represent optical free-space paths, while black dashed lines represent electrical wires. OC: optical coupler; BPF: band-pass filter; PD: photodetector; LPF: low-pass filter. The repetition frequency of comb1 and comb2 are loosely locked to 57.2005 MHz and 57.2000 MHz.

Figure 2 shows the experimental setup of the dual-comb gas detection system. The repetition frequencies of the measurement comb and LO comb are $f_{r1}=57.2005$ MHz and $f_{r2}=57.2000$ MHz, with a repetition frequency difference of $\Delta f_{r}=f_{r1}-f_{r2}=500$ Hz. The outputs of two combs are combined together and interrogate a 16.5 cm long fiber-coupled absorption cell filled with 100 Torr (13.2 kPa) H^13^C^14^N gas (Wavelength References Inc, Corvallis, OR, USA). Another empty cell is put into the reference path to remove the background comb spectrum and obtain an accurate transmittance spectrum. Optical band-pass filter (1557 nm center wavelength, 8 nm FWHM) is used to satisfy band-pass sampling requirement $\Delta \upsilon <f_{r}^{2}/2\Delta f_{r}$, electronic low-pass filter (22 MHz cut-off frequency) is used to suppress the independent response of two combs. Finally, measurement and reference IGMs are generated by two InGaAs photodetectors (PDA05CF2, Thorlabs, Newton, NJ, USA) and sampled by a 16-bit digitizer (M4i.4420-x8, Spectrum, Grosshansdorf, Germany).

**Figure 3:**
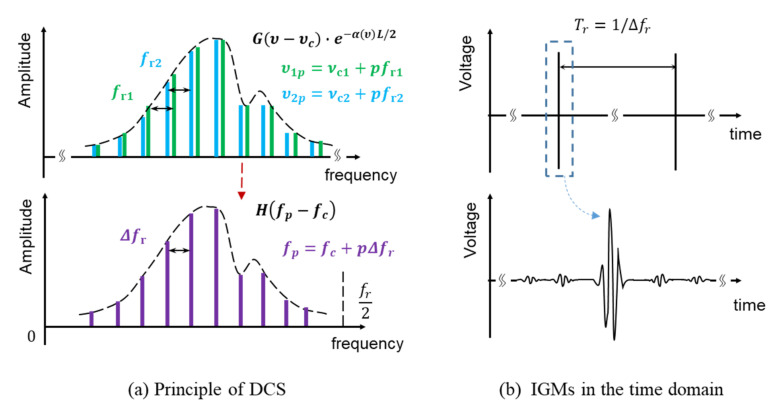
Schematic of DCS principle and interferograms (IGMs) signal. (**a**) Dual-comb down-conversion process. Optical frequency modes of two OFCs (blue and green) beat reciprocally to generate radio frequency (RF) frequency components (purple) according to a mode-to-mode heterodyne interference. (**b**) Typical time-domain IGMs. The carrier-envelope pulse sequence is the inverse Fourier transform of discrete RF magnitude spectrum, while each frame is the inverse Fourier transform of continuous spectrum curve function.

The principle of dual-comb spectroscopy is shown in Figure 3. Measurement comb and LO comb sample the band-pass filter limited normalized curve $G\left(\nu -\upsilon_{c}\right)$ in the frequency domain. According to the Lambert–Beer law, the normalized magnitude spectral curve turns into $G\left(\nu -\upsilon_{c}\right)e^{-\alpha \left(\upsilon \right)L/2}$ after interrogating the gas cell. As the multiheterodyne interference effect of DCS [11,38], the optical frequency modes down-convert to the RF domain carrying the encoded spectral information. The relationship between RF mode $f_{p}$ and optical mode $\upsilon_{p}$ can be expressed as

(1) $$\upsilon_{p}=\frac{\left(f_{p}-f_{c}\right)\left(f_{r1}+f_{r2}\right)}{2\Delta f_{r}}+\upsilon_{c},$$

where $\upsilon_{c}$, $f_{c}$ are the center frequency of optical spectrum and RF spectrum, respectively. The obtained RF spectral curve after down-conversion can then be derived by

(2) $$H\left(f_{p}-f_{c}\right)=G^{2}\left(\upsilon_{p}-\upsilon_{c}\right)e^{-\alpha \left(\upsilon_{p}\right)L}.$$

Then, time-domain IGMs $U\left(t\right)$ obtained by the photodetector is the combination of tens of thousands of RF modes and expressed as

(3) $$U\left(t\right)\;\propto \underset{p}{\sum }H\left(f_{p}-f_{c}\right)\;\cos\left(2\pi f_{p}t+\varphi_{0}\right),$$

which could also be depicted by the carrier-envelope pulse sequence

(4) $$U\left(t\right)\;\propto \underset{N}{\sum }h\left(t-NT_{r}\right)\;\cos\left(2\pi f_{c}t+\varphi_{c}\right),$$

where $h\left(t\right)$ is the inverse Fourier transform of $H\left(f\right)$ and N corresponds to the order of segmented IGMs period. In our experiment, 60 μs observable time window around each centerburst was taken as a frame and real-time spectrum can be obtained by Fourier transform. The time interval between adjacent frames was 2 ms, corresponding to a 500 Hz acquisition rate for real-time spectral analysis.

For a realistic, free-running DCS configuration that suffered from environmental perturbation, the center frequency $f_{c}$ and mode spacing $\Delta f_{r}$ would constantly drift. Due to the large amplification factor between optical frequency $\upsilon_{c}$ and repetition frequency $f_{r}$ (~10^6^ level), center frequency jitter is dominant compared to repetition frequency difference jitter. In addition, since the intrinsic short-term stability of the free-running mode-locked lasers, frequency jitter within each short frame can be neglected compared to the one between different frames [12,38]. Hence, the spectrum of a single frame can be roughly assumed as high fidelity with merely overall center frequency drift, and it is rather easier to correct the spectra directly in the frequency domain instead of dealing with IGMs stream in the time domain.

To align each spectrum frame by frame, center frequency variation $\delta f_{N}$ should be accurately estimated. First, reference and measurement IGMs frames are respectively Fourier transformed to obtain real-time normalized spectra $H_{N}^{ref}\left(f-f_{c}-\delta f_{N}\right)$ and $H_{N}^{mea}\left(f-f_{c}-\delta f_{N}\right)$. Reference spectra represent the background spectral function without gas absorption, then real-time transmission spectra can be calculated by

(5) $$T_{N}\left(f-f_{c}-\delta f_{N}\right)=\frac{H_{N}^{mea}\left(f-f_{c}-\delta f_{N}\right)}{H_{N}^{ref}\left(f-f_{c}-\delta f_{N}\right)}.$$

In this way, fluctuated dual-comb background spectrum can be greatly removed regardless of the spectral curve shape.

**Figure 4:**
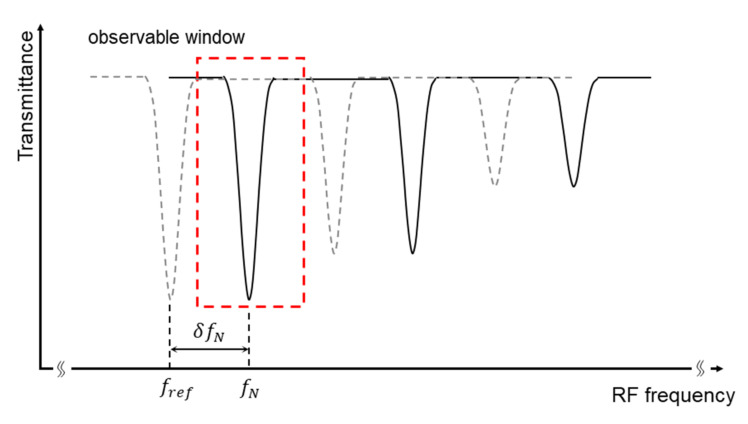
Diagram of center frequency variation extraction method. As center frequency jitter is dominant compared to repetition frequency difference jitter, real-time transmittance spectra can be assumed as high fidelity with merely overall center frequency drift. To estimate center frequency variation $\delta f_{N}$ frame by frame, 45 kHz observable window can be isolated around a specific absorption line. After removing the residual baseline, the drift of the center of gravity within each observable window can be recorded as $\delta f_{N}$. In this figure, center frequency variation is exaggerated for illustrative purposes.

Second, as shown in Figure 4, observing windows around a specific absorption line covering 45 kHz (~50 GHz in the optical domain) are isolated, polynomial fit can be imposed upon the edge of the observing window to remove the residual spectral baseline [13,29]. Then, the center of gravity (COG) of the isolated absorption line $f_{N}$ can be calculated frame by frame. Expected center frequency of specific absorption line $f_{ref}$ can be freely set around actual drift range, e.g., absorption line center of the first frame $f_{1}$. The variation of overall frequency axis can be recorded as $\delta f_{N}=f_{N}-f_{ref}$. Therefore, the frequency axis of each transmittance spectrum is supposed to be recalibrated by

(6) $$T_{N}\left(f-f_{c}\right)=T_{N}\left(f-f_{c}-\delta f_{N}+\delta f_{N}\right).$$

The above-mentioned frequency axis alignment method guarantees the consistency of real-time transmittance spectra, which enables the following spectrum averaging to achieve better sensitivity as:

(7) $$\bar{T}\left(f-f_{c}\right)=\frac{\sum_{N}T_{N}\left(f-f_{c}\right)}{N}.$$

Finally, similar to other computational post-processing methods without accurate optical reference [31,34,36], our method is not capable of measuring the absolute frequency of OFC modes. To realize accurate frequency up-conversion from RF domain to optical frequency domain, prior absorption line position of H^13^C^14^N molecular $\upsilon_{\mathrm{ref}}$ is marked as a frequency reference. Then, considering the scale transformation relationship between RF components and optical elements, the whole optical frequency axis υ can be accurately reconstructed by

(8) $$\upsilon =\frac{\left(f-f_{ref}\right)\left(f_{r1}+f_{r2}\right)}{2\Delta f_{r}}\upsilon_{ref}.$$

## 3. Results

**Figure 5:**
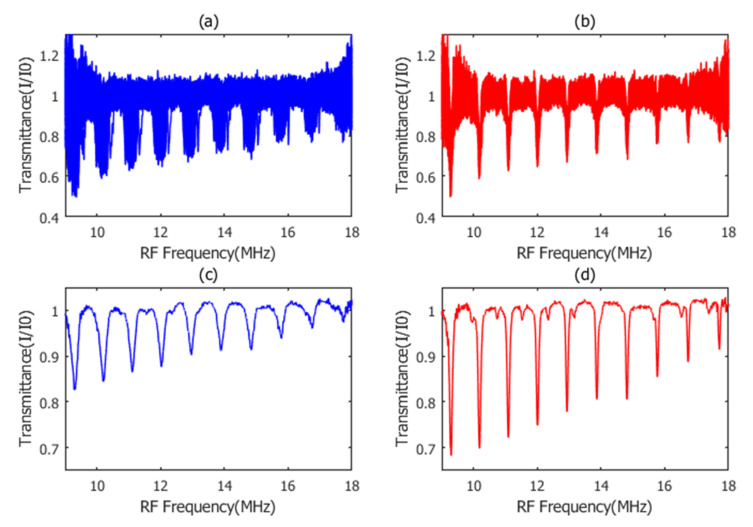
The effect of our periodic spectrum alignment method for 0.2 s transmittance averaging. The baseline has been normalized by fitting. (**a**) Raw transmittance spectra of 100 different IGMs frames; (**b**) aligned transmittance spectra of 100 different IGMs frames; (**c**) averaged raw transmittance spectra of 100 different IGMs frames, the relative center frequency drift between them prominently broadens the linewidth and distort the lineshape; and (**d**) averaged transmittance spectra of 100 aligned IGMs frames by periodic spectrum alignment method.

In our experiment, the amplitude information of over 18800 optical modes (~1.1 THz spectrum range) was simultaneously obtained within 60 μs acquisition time. The spectral measurement range can be further extended by adjusting the center wavelength of an optical band-pass filter. In terms of real-time spectral performance, the figure of merit of DCS configuration is ~0.34 × 10^7^ Hz^1/2^, which is comparable with other more complicated schemes [11]. The effectiveness of our periodic spectrum alignment method is shown in Figure 5. Slow baseline has been normalized by polynomial fitting [13,29]. For long acquisition time, the raw transmittance spectra of different frames are suffered from ~0.5 MHz center frequency drift, which would cause severe linewidth to broaden and lineshape distortion after 0.2 s spectrum averaging. In contrast, the periodic spectrum alignment method significantly suppresses center frequency jitter and circumvents extra absorption linewidth broaden, increasing the measurement resolution and sensitivity.

**Figure 6:**
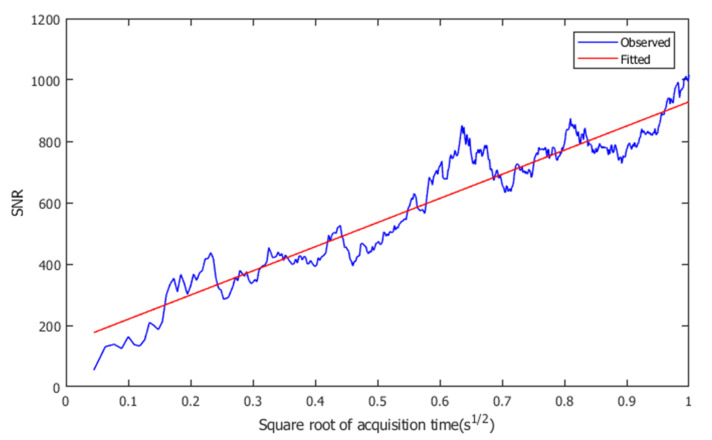
Signal noise ratio (SNR) improvement with the increase of averaging time. The blue line represents observed SNR, while the red line is the linear fit result. It is verified that the relationship between the square root of acquisition time and SNR is linear, and the SNR would keep increasing for longer acquisition time.

To investigate the effect of transmittance spectrum averaging on a longer time scale, the line relationship between the root mean square of acquisition time and signal noise ratio (SNR) is shown in Figure 6. Due to the power attenuation in the wings of comb spectra, the noise level would increase when shifting away from the center frequency. In this paper, we define the SNR as the inverse of averaged noise standard deviation in a transmittance spectrum. In our experiment, the averaged noise standard deviation is calculated after removing 45 kHz frequency windows around each line. The standard deviation of the remaining transmittance spectrum is regarded as the averaged noise standard deviation. After 1 s spectrum averaging, the SNR is improved from 54 to 1017 compared to single frame measurement, and there is no sign that SNR increasing trend is about to break down over a longer time scale. Therefore, SNR can be further increased by extending the measurement time.

**Figure 7:**
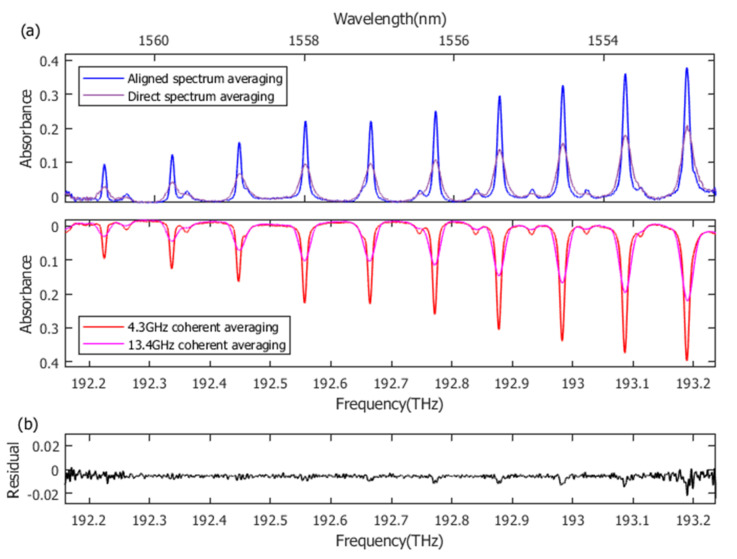
Absorbance spectrum over 1 s averaging. (**a**) Resolution improvement effect of aligned spectrum averaging compared to direct spectrum averaging. Meanwhile, our measured absorbance spectrum shows a great agreement with the result obtained by conventional optical reference error correction with 4.3 GHz resolution after 1 s coherent averaging, proving the practicality of our method for gas detection applications. (**b**) Residual between absorbance spectrum obtained by our method and conventional error correction with 4.3 GHz resolution. The standard deviation is ~0.002 over the 1.1 THz spectral range.

To verify the actual spectrum measurement performance, the conventional optical referencing error correction method [36,44] was executed simultaneously as a comparison. Two CW lasers (ORION, RIO, Santa Clara, CA, USA), located at ~1550.148 nm and ~1564.652 nm, were used as intermedium to obtain relative beat between dual combs. Time jitter and carrier-envelope phase jitter can then be extracted continuously. After time-domain phase rotation and resample, 1 s coherent averaging in the time domain is available for all frames within the IGMs stream. This conventional error correction method can achieve a mode-resolved spectrum with at most 57.2 MHz resolution, which is more than sufficient to measure intrinsic absorption lineshape with GHz level width. However, the physical resolution of our simplified aligned spectrum averaging method is limited by residual jitter within each frame. Thus, the measured lineshape of low-pressure (100 Torr) H^13^C^14^N gas is the convolution of intrinsic absorption lineshape and instrumental lineshape. To match the actual resolution of our method, apodized Hanning window can be imposed upon coherent averaged IGM to obtain artificially decreased resolution for conventional mode-resolved measurement. Absorbance spectrum is derived from transmittance spectrum after 1 s averaging, which is more widely used in actual gas detection applications. As is shown by Figure 7, 1 s averaged absorbance spectrum by our method is in great agreement with the one by conventional error correction method with 4.3 GHz (~0.14 cm^−1^) resolution. Several dominant absorption lines and weak hot-band transitions in the P branch of H^13^C^14^N are clearly distinguished over 1.1 THz spectral widths. The fluctuation range of residual is within 0.026 and the overall standard deviation is ~0.002. Systematic residual error around absorption lines can be attributed to the lineshape difference between the spectrum averaging method (determined by residual frequency noise) and the time-domain error correction method (determined by apodized Hanning window). In contrast, direct spectrum averaging without periodic alignment broadens the absorption line and can achieve only 13.4 GHz resolution. The resolution improvement effect of our method is remarkable, ~4.3 GHz physical resolution is comparable with the commercial near-infrared optical spectrum analyzer, which is already sufficient for numerous gas detection applications. Our method dispenses with bulk optical reference configuration or complex computational algorithm, offering a cost-effective error correction method for dual-comb gas detection.

## 4. Discussion

In this paper, we demonstrate a simple and effective frequency-domain post-processing method to suppress center frequency drift and improve spectral resolution. It should be noticed that our method requires sufficient SNR around chosen absorption line to accurately estimate the center frequency drift in the spectral range. Once the first real-time spectrum is obtained, we can detect all the absorption lines and compare their SNR to make an optimal decision automatically. The performance of obtained absorbance spectrum based on different reference absorption lines are shown in the Supplementary Materials (Figure S1 and Table S1). The SNR around chosen absorption line (defined as line’s height multiplied by the inverse of noise standard deviation) as low as 6 can still enable accurate alignment. If all absorption lines of real-time spectrum are immerged in noise, the SNR of dual-comb spectroscopy configuration should be further improved by using photodetector with larger dynamic range [11], narrow-band filtering [29], fiber pool chirping [36], multi-pass cell [32], or enhanced cavity [35].

Another key factor of our experiment is the Fourier transform calculation resolution. To calculate center frequency with high accuracy, sufficient sampling points within a single absorption line window should be guaranteed. For each IGMs frame, a zero-padding process was utilized to make calculation resolution (~50 MHz) much lower than physical resolution (~4.3 GHz), ensuring the accuracy of COG estimation. Actually, the zero-padding process could also be replaced by regional interpolation or nonlinear fit to reduce the calculation cost of Fourier transform.

As a one-step correction method in the frequency domain, our alignment method only compensates for center frequency jitter frame by frame. The alignment method based on global cross-correlation [43] was also adopted in the experiment and achieved consistent performance with our method (shown in Figure S2 in the Supplementary Materials). The ultimate physical resolution limitation is mainly due to the center frequency jitter within each frame. As the stretch of the frame duration, true center frequency jitter far away from the centerburst would deviate from the measured value, which is analogous to imposing an apodized window upon each IGM frame. On the other hand, uncompensated repetition frequency jitter would also be non-negligible when the spectral measurement range is extremely large. In this situation, the wing of the comb spectrum would suffer from resolution degradation. However, spectral resolution in our experiment is comparable with a commercial near-infrared optical spectrum analyzer, which is already sufficient for numerous Doppler-limited molecular spectrum analysis applications in environment monitor, process control, and combustion diagnosis. If higher resolution is required for some specific applications, relatively complicated analog adaptive sampling [34] or digital error correction [36] are alternatives to completely compensate time jitter and center frequency jitter.

In this paper, hydrogen cyanide gas detection was demonstrated as a proof of principle experiment. In the near-infrared region determined by erbium-doped fiber frequency comb, acetylene [27], methane [45], ammonia [32], etc. can be easily detected with a large absorption coefficient. Since our method is not limited by spectral area, the spectral coverage can be broadened further to nearly one octave using highly nonlinear fiber [4,46], enabling multicomponent analysis and quantitative measurement. In addition, difference frequency generation (DFG) and optical parametric oscillator (OPO) technique [5] can be used to convert the spectral coverage to mid-infrared molecular fingerprint region, where gas molecules tend to have stronger characteristics.

Our spectrum alignment algorithm is not limited to low repetition frequency fiber DCS, it can also benefit other configurations such as microresonator soliton DCS [47,48] to suppress possible resolution degradation. Considering the simplicity, real-time implementation potential, and free of external CW laser of our method, it creates a bright perspective for highly integrated field-deployed DCS applications in the future.

## 5. Conclusions

We propose a frequency domain spectrum alignment and averaging method to improve the resolution and sensitivity of quasi free-running dual-comb spectroscopy. Neither specially designed delicate resonant cavity configuration nor complicated multi-jitter time-domain correction scheme are used to realize Doppler-limited molecular absorption spectrum measurement. By tracking center frequency drift frame by frame, a common dual-comb gas detection system is greatly simplified, while long-time spectrum averaging is still available to enhance the SNR. In the experiment, the H^13^C^14^N absorbance spectrum is measured with a ~0.002 standard deviation and 4.3 GHz resolution across over 1.1 THz spectral range. Our method has the potential to implement on real-time process platforms such as FPGA, providing a cost-effective alternative tool for field-deployed DCS applications.

## Data Availability

Not applicable.

## Supplementary Materials

The following are available online at https://www.mdpi.com/1424-8220/21/3/903/s1, Figure S1: Performance of our alignment method using different reference absorption lines, Table S1: The standard deviation of residual using different absorption lines, Figure S2: Performance comparison between our method and global cross-correlation.
