# Supplementary material for: Improving Resolution of Dual-Comb Gas Detection Using Periodic Spectrum Alignment Method
Source: Sensors (Basel). 2021 Jan 29;21(3):903. doi: 10.3390/s21030903 (PMC7866286; doi:10.3390/s21030903)
Supplement: Supplementary file 1 [file sensors-21-00903-s001.pdf]

sensors

Supplementary Information for Improving Resolution of Dual-comb Gas Detection Using Periodic Spectrum Alignment Method

Haoyang Yu [1], Qian Zhou [1], Xinghui Li [1], Xiaohao Wang [1], Xilin Wang[2] and Kai Ni [1,*]

[1] Division of Advanced Manufacturing, Tsinghua Shenzhen International Graduate School, Tsinghua University, Shenzhen, 518055, China;
yu-hy16@mails.tsinghua.edu.cn; zhou.qian@sz.tsinghua.edu.cn; li.xinghui@sz.tsinghua.edu.cn; wang.xiaohao@sz.tsinghua.edu.cn;

[2] Engineering Laboratory of Power Equipment Reliability in Complicated Coastal Environments, Tsinghua Shenzhen International Graduate School, Tsinghua University, Shenzhen, 518055, China;
wang.xilin@sz.tsinghua.edu.cn;

* Correspondence: ni.kai@sz.tsinghua.edu.cn;

Citation: Yu, H.; Zhou, Q.; Li, X.; Wang, X.; Wang, X.; and Ni, K. Improving Resolution of Dual-Comb Gas Detection Using Periodic Spectrum Alignment Method. *Sensors* **2021**, *21*, 903. https://doi.org/10.3390/s21030903

Received: 30 November 2020
Accepted: 27 January 2021
Published: 1 February 2021

Publisher's Note: MDPI stays neutral with regard to jurisdictional claims in published maps and institutional affiliations.

Copyright: © 2021 by the authors. Licensee MDPI, Basel, Switzerland. This article is an open access article distributed under the terms and conditions of the Creative Commons Attribution (CC BY) license (http://creativecommons.org/licenses/by/4.0/).

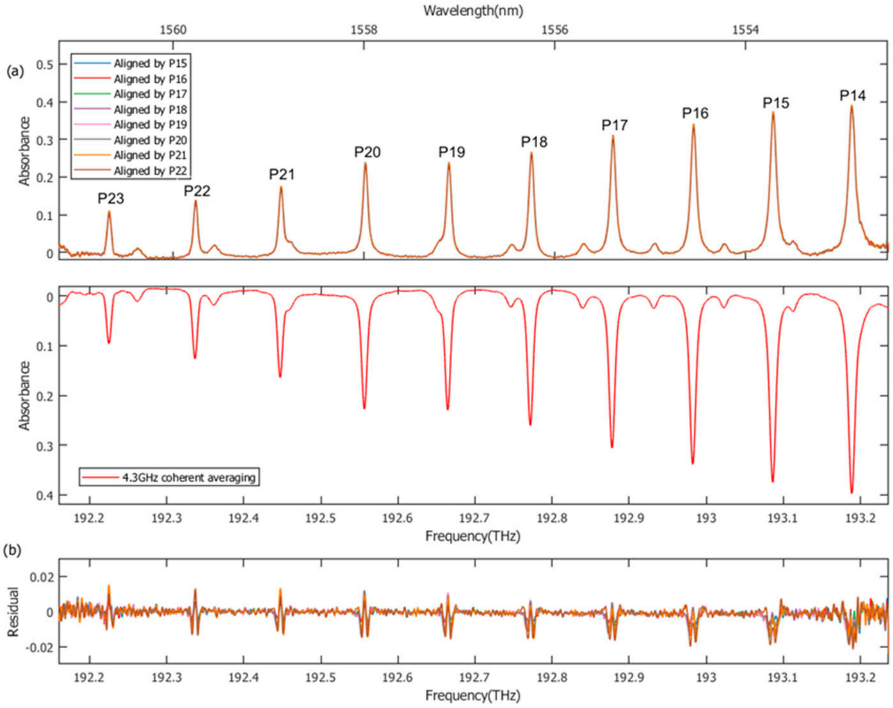

Figure S1. Absorbance spectrum over 1 s averaging based on alignment by different reference absorption lines. (a) Direct comparison of aligned spectrum by P15-P22 lines. Except the two absorption line at the edge of the comb spectrum which are greatly masked by noise, the obtained absorbance spectra using all of the 8 different reference lines show great agreement with the result obtained by conventional optical reference error correction with 4.3 GHz resolution after 1 s coherent averaging, proving the practicality of our method using different reference lines. (b) Residual between obtained absorbance spectra and conventional error correction.

Table S1. The standard deviation of residual between obtained absorbance spectra using different absorption lines and conventional error correction. The standard deviation of residual is merely ~0.004 even in the case of maximum deviation.

Line	The standard deviation of residual
P15	0.0020
P16	0.0025
P17	0.0028
P18	0.0031
P19	0.0034
P20	0.0033
P21	0.0034
P22	0.0040

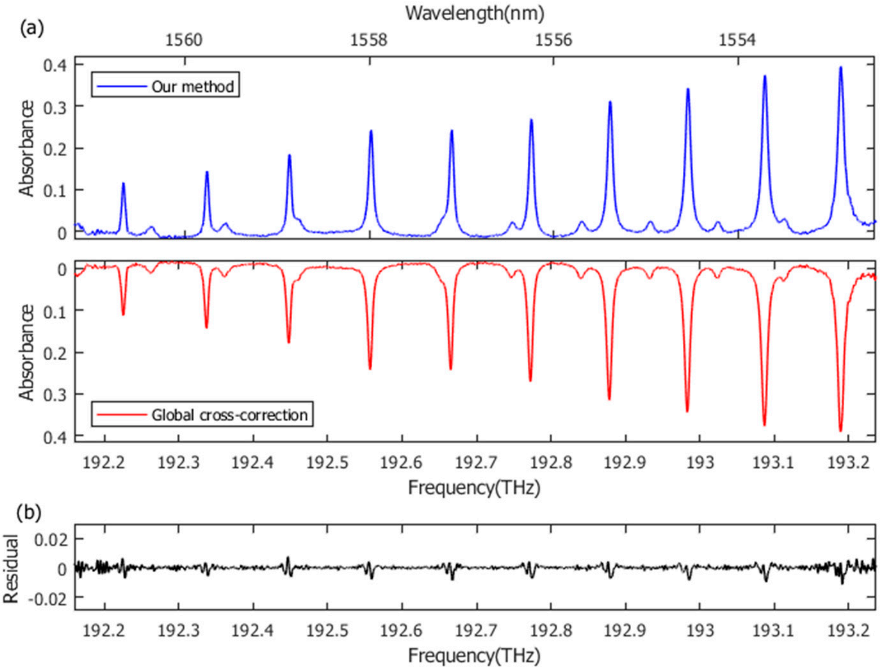

Figure S2. Absorbance spectrum over 1 s averaging based on global cross-correlation method [1] and our regional COG algorithm. The standard deviation of residual between two methods is ~0.0019. The systematic error around absorption lines is mainly due to the influence of amplitude noise. However, the performance difference between two methods is considerably small.

References

1. Kara, O.; Zhang, Z.; Gardiner, T.; Reid, D.T. Dual-comb mid-infrared spectroscopy with free-running oscillators and absolute optical calibration from a radio-frequency reference. Opt. Express 2017, 25, 16072-16082.
